# Bismuth-Decorated Honeycomb-like Carbon Nanofibers: An Active Electrocatalyst for the Construction of a Sensitive Nitrite Sensor

**DOI:** 10.3390/molecules28093881

**Published:** 2023-05-04

**Authors:** Fengyi Wang, Ye Li, Chenglu Yan, Qiuting Ma, Xiaofeng Yang, Huaqiao Peng, Huiyong Wang, Juan Du, Baozhan Zheng, Yong Guo

**Affiliations:** 1 Institute of Quality Standard and Testing Technology for Agro-Products, Sichuan Academy of Agricultural Sciences, Chengdu 610066, China; wangfengyichem@163.com; 2 College of Chemistry, Sichuan University, No. 29 Wangjiang Road, Chengdu 610064, China; liye7198@163.com (Y.L.); ma157308@163.com (Q.M.); guoy@scu.edu.cn (Y.G.); 3 Key Laboratory of Aviation Fuel & Chemical Airworthiness and Green Development, The Second Research Institute of Civil Aviation Administration of China, Chengdu 610041, China; ycl1771613522@foxmail.com (C.Y.); penghuaqiao@fccc.org.cn (H.P.); 4 College of Chemistry and Chemical Engineering, Henan Normal University, Xinxiang 453002, China; hywang@htu.edu.cn

**Keywords:** nitrite sensor, honeycomb-like carbon nanofibers, Bi nanoparticles, nitrite detection

## Abstract

The existence of carcinogenic nitrites in food and the natural environment has attracted much attention. Therefore, it is still urgent and necessary to develop nitrite sensors with higher sensitivity and selectivity and expand their applications in daily life to protect human health and environmental safety. Herein, one-dimensional honeycomb-like carbon nanofibers (HCNFs) were synthesized with electrospun technology, and their specific structure enabled controlled growth and highly dispersed bismuth nanoparticles (Bi NPs) on their surface, which endowed the obtained Bi/HCNFs with excellent electrocatalytic activity towards nitrite oxidation. By modifying Bi/HCNFs on the screen-printed electrode, the constructed Bi/HCNFs electrode (Bi/HCNFs-SPE) can be used for nitrite detection in one drop of solution, and exhibits higher sensitivity (1269.9 μA mM^−1^ cm^−2^) in a wide range of 0.1~800 μM with a lower detection limit (19 nM). Impressively, the Bi/HCNFs-SPE has been successfully used for nitrite detection in food and environment samples, and the satisfactory properties and recovery indicate its feasibility for further practical applications.

## 1. Introduction

With the development of modern technology, people are increasingly aware of the importance of food safety for health because the abuse of common food additives, such as nitrite, can bring a great threat to human health [1,2]. On the one hand, the excessive uptake of nitrite can react with amines under acidic conditions and form nitrosamine carcinogens, which will result in cancer, hypertension and neurodegenerative diseases [3,4]. In addition, nitrite can also interact with hemoglobin, and the generated methemoglobin can inhibit the ability of blood to carry oxygen, leading to serious methemoglobinemia [5]. On the other hand, nitrite, as a common water-soluble nitrogenous compound, is ubiquitous within the natural environment, which can lead to water pollution and eutrophication during nitrogen cycling [6]. Notably, the World Health Organization (WHO) has stipulated that the maximum limit of nitrite concentration is 3 mg L^−1^ in drinking water [7]. Therefore, it is urgent to develop effective analytical techniques for nitrite identification and precise quantitative detection.

In the process of the literature survey, we found that published research papers increased gradually from 2018 to the present when searching for “nitrite sensor” on the Web of Science, and methods based on chemiluminescence [8], fluorescence [9,10], ion chromatography [11], colorimetric method [12] and electrochemistry [1,13,14] have been explored extensively for nitrite detection, while most of these methods usually suffer from needing relatively expensive instruments, time-consuming operation processes and toxic chemical reagents, which greatly hinder their wide applications. Compared with these methods, electrochemical technology has been proven to be a promising method because it can be easily miniaturized and constructed cost-effectively. It is well known that the composition and morphology of catalyst materials can greatly influence the performance of electrochemical sensors, including their sensitivity, selectivity, stability and reproducibility, etc. Therefore, the design and preparation of high-performance electrocatalysts is the premise for constructing electrochemical sensors for nitrite detection with excellent properties.

Our previous research has demonstrated that Bi-based materials possess a high affinity for nitrogen, which therefore greatly boosts the nitrogen reduction rate [15,16]. Thus, these Bi-based materials have been widely used as electrocatalysts to catalyze the reduction of nitrite and nitrate to generate ammonia [17]. Inspired by these, we speculate that Bi-based nanomaterials will also have good performance in constructing electrochemical sensors for nitrite detection, but there are few reports in this area. Nevertheless, since Bi is a metal with semiconducting properties, its poor electrical conductivity will impede its further applications in electrochemistry [18]. To overcome this disadvantage, conductive carbon materials (such as carbon nanofibers [19], carbon nanotubes [20,21], graphene [22,23], biomass carbon [24], etc.) have been selected as the substrates, and they can greatly improve the intrinsic electrical conductivity of Bi-based nanocomposites. Therefore, nanofibers have been extensively used in biomedical and antibacterial fields because of their low toxic, biocompatible and biomimetic properties [25,26]. In this context, porous carbon nanofibers (PCNFs) with high conductivity and large specific surface area have been the optimal choice to support and distribute Bi nanoparticles [1]. To date, PCNFs have generally been fabricated using corrosive chemical agents such as KOH or HNO_3_, yet they are unsafe and not environmentally friendly in large-scale industrial manufacturing [27,28]. In terms of the template method, the main route of conventional synthesis relies on sacrificial polymers but they do not have an easily controlled pore size. Therefore, how to increase the porosity and conductivity of carbon nanofibers simultaneously as well as keeping their integrity is still deemed a great challenge [29,30].

**Scheme 1:**
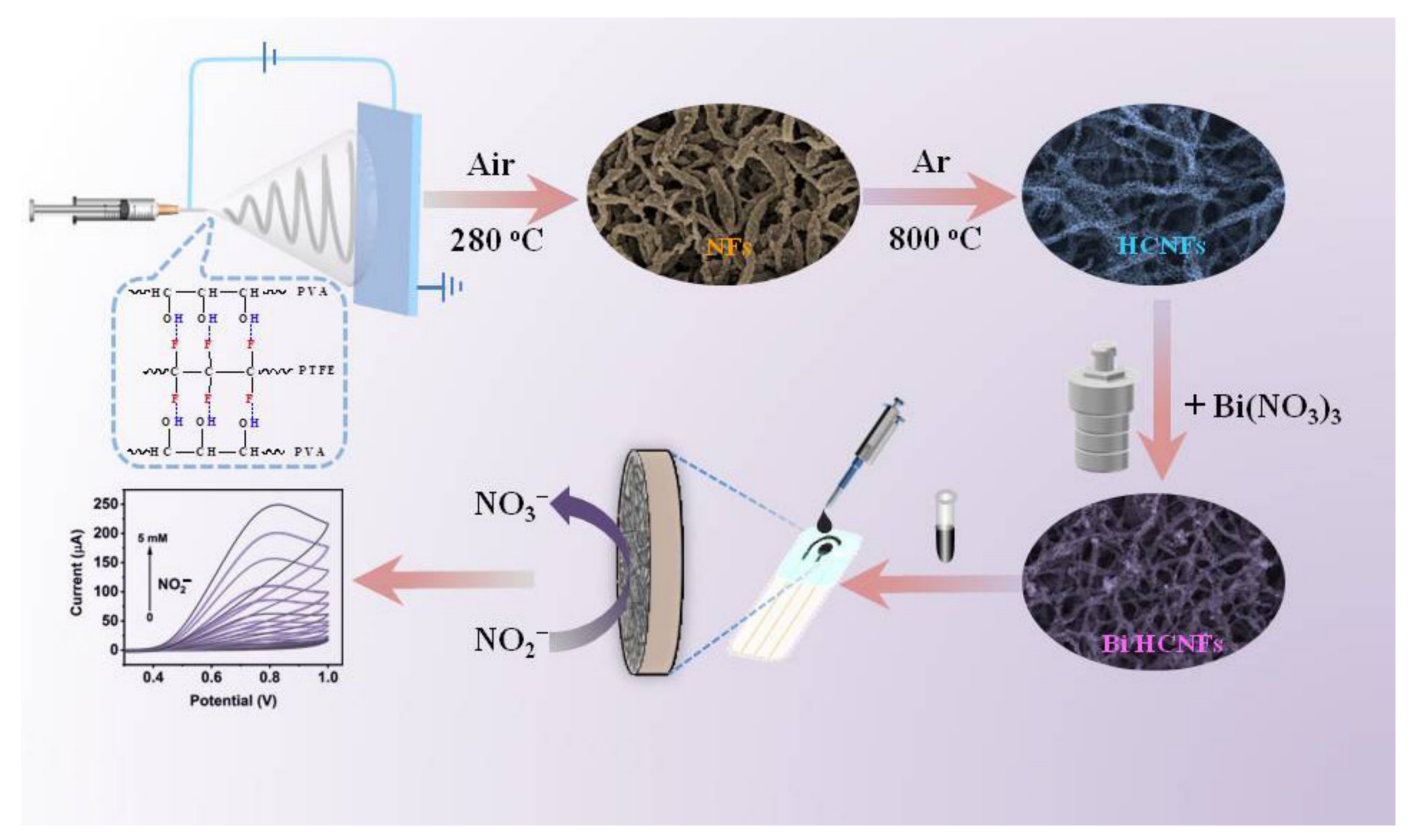
Schematic illustration of Bi/HCNFs-SPE construction and its electrochemical response to nitrite.

Herein, one-dimensional (1D) carbon nanofibers were firstly prepared using polyvinyl alcohol (PVA) and polytetrafluoroethylene (PTFE) as precursors using electrospinning technology (Scheme 1), and the decomposition of PTFE during the carbonization process under Ar atmosphere could lead to the formation of honeycomb-like carbon nanofibers (HCNFs). The specific porous structure of carbon nanofibers could result in the uniform growth of Bi nanoparticles with smaller sizes on their surface and the successful preparation of Bi/HCNFs nanocomposite, which has not been reported before. By decorating Bi/HCNFs on the screen-printed electrode (SPE), the constructed Bi/HCNFs-SPE exhibits excellent electrocatalytic properties for nitrite oxidation, and can be used for nitrite detection in one drop of aqueous with higher sensitivity, low detection limit, preferable selectivity and long-term stability. Importantly, this electrochemical strategy based on Bi/HCNFs-SPE has also been successfully employed for nitrite determination in real food and environmental samples, and the desired results manifest that this proposed electrochemical sensor could work as a fulfilling sensing platform for a highly sensitive analysis of nitrite in the food industry and natural environment.

## 2. Results and Discussion

### 2.1. Synthesis and Characterization of Bi/HCNFs

**Figure 1:**
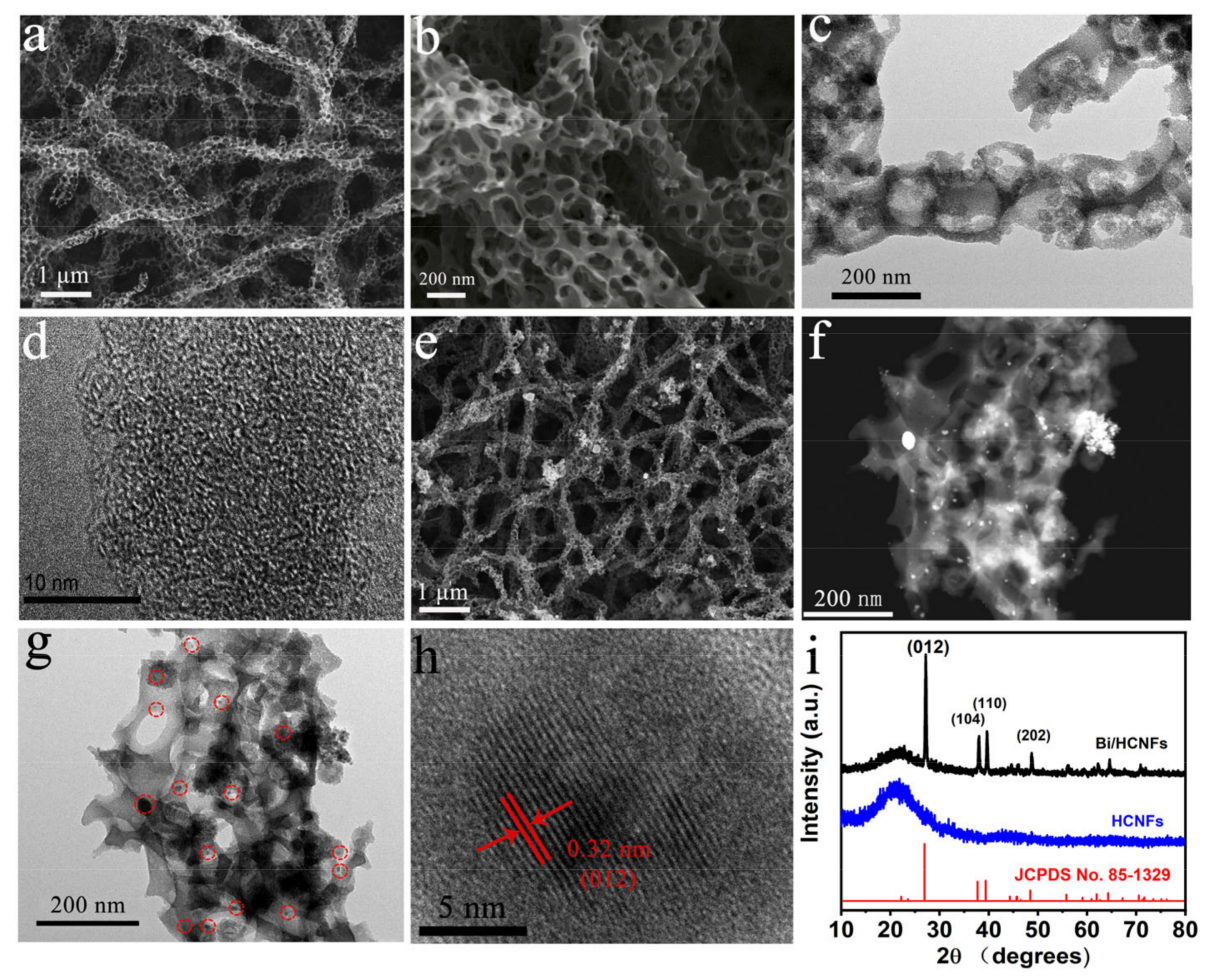
SEM (**a**,**b**), TEM (**c**) and HR-TEM (**d**) images of HCNFs; SEM (**e**), TEM (**f**,**g**) and HR-TEM (**h**) images of Bi/HCNFs, in which the red cycle and the distance of red arrow are refer to Bi NPs and its interplanar spacing; (**i**) XRD patterns of HCNFs and Bi/HCNFs.

As shown in Scheme 1, polymer nanofibers were first prepared with an electrospinning method using PVA and PTEE as precursors. After introducing PTFE into the PVA aqueous solution, the formed reversible hydrogen bond between PVA and PTFE could act as an end-capping agent to regulate the crosslinking degree of viscous solution, benefiting the production of uniformly stable nanofibers (NFs). The as-spun fibers were pretreated in an air atmosphere at 280 °C to prevent PVA pyrolysis and to stabilize their 1D nanostructure without collapsing during the pyrolysis process; the color of the fibers changed to brown in this procedure. During the process of annealing at 800 °C for 2 h, the carbonization of PVA formed a carbon skeleton and the decomposition of PTFE could create continuous macropores in carbon nanofibers, which led to the honeycomb-like structure of carbon nanofibers (HCNFs). Then, the zeta-potential of HCNFs’ dispersion was tested to −10.15 mV. Using HCNFs as the substrate, Bi^3+^ could be electrostatically adsorbed on the HCNFs with a negative charge, and subsequently could be reduced to a zerovalent Bi atom and grown controllably on its surface through a hydrothermal method, thus finally forming Bi/HCNFs nanocomposites. In this process, the added hydrazine could not only act as a reducing agent to reduce Bi^3+^ ions, but also yield N_2_ gas to prevent the re-oxidation of Bi NPs in solution. Given the specific surface area of HCNFs, as well as the high affinity of Bi-based materials on nitrogenous compounds, we speculated that the prepared Bi/HCNFs nanocomposite would exhibit high electrocatalytic activity for nitrite oxidation. Figure 1a–d show the SEM and TEM images of the as-fabricated HCNFs; it can be seen that the HCNFs are composed of a honeycomb skeleton with homogeneous and interlinked hollow holes over the fiber. The high-resolution TEM (HR-TEM) of Figure 1d further illustrates the amorphous property of HCNFs with disordered graphitic ribbons [31]. The SEM and TEM images of Bi/HCNFs were displayed in Figure 1e–h. From Figure 1e, we can see the presence of Bi NPs on the HCNFs’ surface compared with that of HCNFs (Figure 1a). Furthermore, the TEM image of Bi/HCNFs (Figure 1f,g) also indicates uniformly dispersed Bi NPs on HCNFs with an average diameter of 6.86 nm (Figure S1), which is much smaller than the reported Bi NPs loaded on CNFs without pores [19], demonstrating that the HCNFs can regulate the distribution of Bi NPs and restrain their growth. As a comparison, Bi NPs were also synthesized without the presence of HCNFs, and the larger particles indicate that the HCNFs were pivotal to the formation of ultrasmall nanoparticles (Figure S2), further proving that the HCNFs could hinder the growth of Bi NPs. These results all demonstrate successfully synthesized honeycomb-like carbon nanofibers with smaller Bi NPs, which might endow higher electrocatalytic activity. Figure 1h shows the HR-TEM image of Bi/HCNFs, and the interlayer spacing of 0.32 nm is attributed to the (012) plane of metal Bi, demonstrating the formed Bi NPs on HCNFs. In addition, the STEM with corresponding energy dispersive X-ray (EDX) elemental mapping images further demonstrated the homogeneously distributed Bi NPs on HCNFs (Figure S3). Figure 1i presents the X-ray diffraction (XRD) patterns of HCNFs and Bi/HCNFs. The distinct diffraction peaks at 27.1°, 37.9°, 39.6°, 48.7° and 64.5° (2θ) corresponded to the (012), (104), (110), (202) and (122) planes of metal Bi (JCPDS No. 85-1329), respectively. The broad diffraction peak at about 23° revealed the defected or disordered structure of HCNFs [32]. Therefore, the XRD results and, above all, characterization data, further proved the successful synthesis of distributed Bi NPs-decorated honeycomb-like carbon nanofibers (Bi/HCNFs).

**Figure 2:**
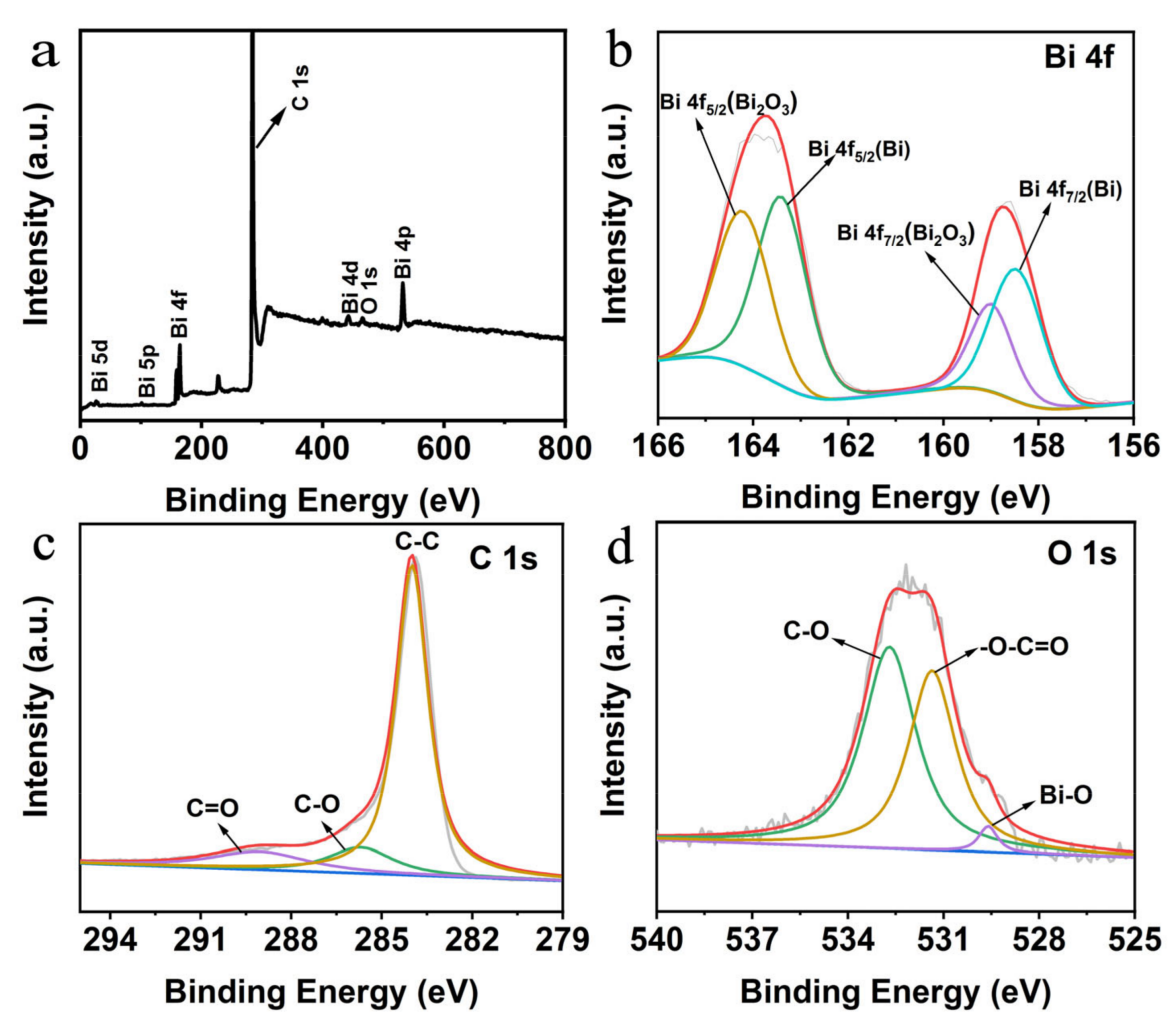
(**a**) XPS survey spectrum of Bi/HCNFs. High-resolution XPS spectrum of Bi 4f (**b**), C 1s (**c**) and O 1s (**d**) of Bi/HCNFs.

The elemental composition and chemical state of Bi/HCNFs were then characterized by X-ray photoelectron spectroscopy (XPS). The survey XPS spectrum shown in Figure 2a indicates the presence of Bi, C and O on Bi/HCNFs. The high-resolution XPS spectra of Bi 4f can be deconvoluted into four peaks (Figure 2b). The peaks located at 164.2 eV and 159.0 eV were assigned to the 4f_5/2_ and 4f_7/2_ (Bi_2_O_3_), respectively. The other two peaks, at 163.4 eV and 158.4 eV, belong to Bi 4f_5/2_ and Bi 4f_7/2_ (Bi), respectively [33]. The emergence of a small amount of Bi_2_O_3_ may be attributed to the oxidation of the as-prepared material [33,34]. The C 1s given in Figure 2c revealed three peaks at 283.9 eV, 285.7 eV and 289.0 eV, corresponding to C-C, C-O and C=O, respectively. The formation of C=O mainly originated from the oxidation of -OH during the annealing process. The O 1s spectrum illustrated in Figure 2d can be divided into three distinct peaks with binding energy centered at 532.7 eV, 531.5 eV and 529.5 eV, which are ascribed to C-O, -O-C=O and Bi-O bonds, respectively. These results further confirmed that Bi NPs are well grown on HCNFs.

### 2.2. Investigation of Electrocatalytic Behavior of Bi/HCNFs toward NO_2_^−^

**Figure 3:**
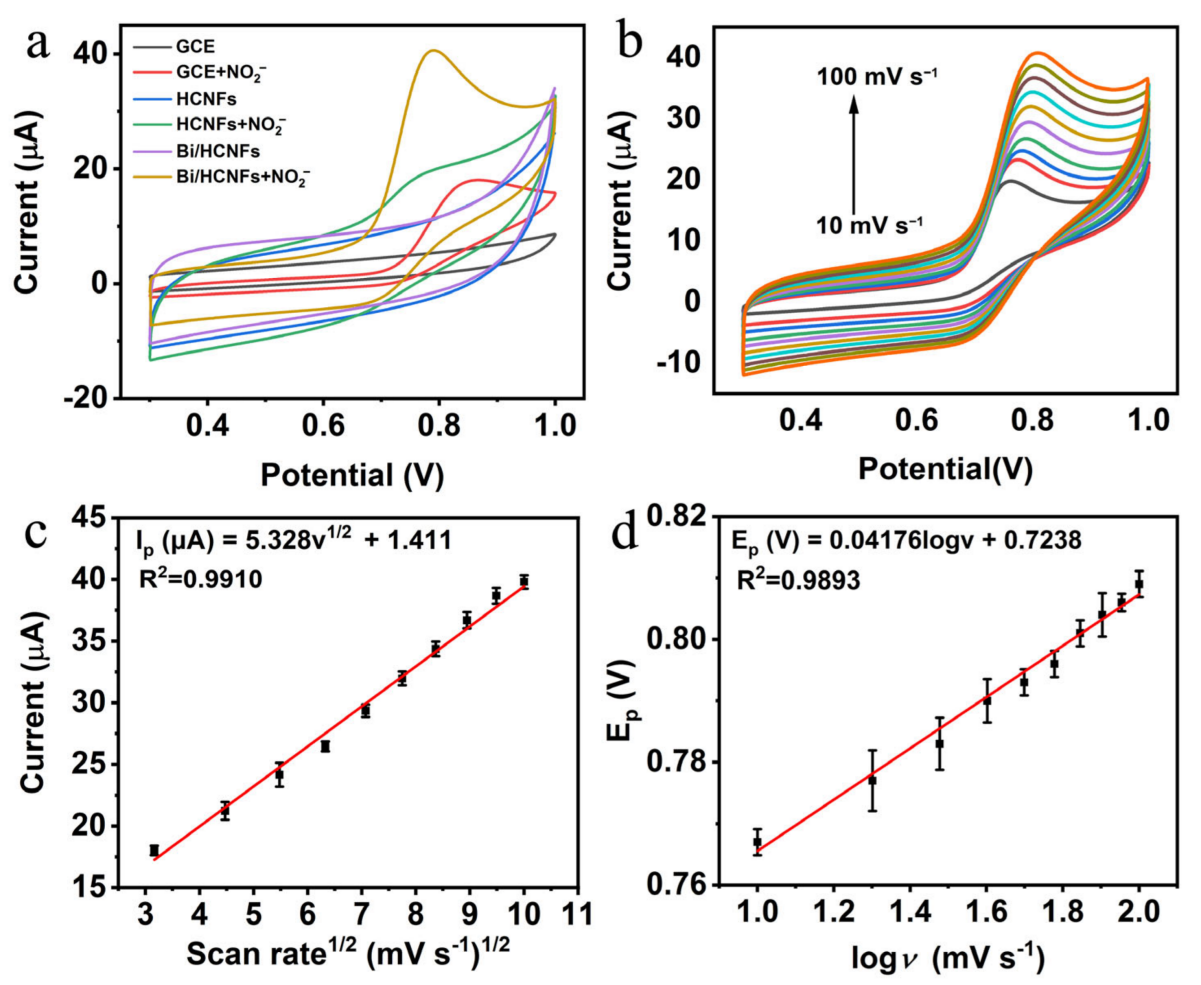
(**a**) CV curves of bare GCE, HCNFs and Bi/HCNFs-modified GCE in 0.1 mol·L^−1^ PBS solution (pH 7.0) before and after the addition of NO_2_^−^ (1 mmol·L^−1^) at a scan rate of 50 mV s^−1^. (**b**) CV curves of Bi/HCNFs in 0.1 mol·L^−1^ PBS solution (pH 7.0) with the presence of NO_2_^−^ (1 mmol·L^−1^) at different scan rates. (**c**) Linear relationship between anodic peak current and the square root of scan rate. (**d**) Plots of the oxidative peak potential versus the logarithm of scan rates.

The electrocatalytic performance of as-prepared Bi/HCNFs towards NO_2_^−^ was investigated by locating Bi/HCNFs on a glassy carbon electrode (GCE). Figure 3a presents the cyclic voltammograms (CVs) of bare GCE, HCNFs/GCE and Bi/HCNFs/GCE in 0.1 mol·L^−1^ PBS (pH 7.0) before and after the addition of NO_2_^−^ (1 mmol·L^−1^). From the results, we can see that all three electrodes had an electrochemical response to NO_2_^−^, and the Bi/HCNFs/GCE had the highest response to NO_2_^−^ oxidation, with an anodic peak at 0.79 V, which was lower than that of bare GCE (0.86 V). The bigger response current of Bi/HCNFs/GCE to NO_2_^−^ than that of HCNFs/GCE also indicated the higher catalytic activity of Bi/HCNFs compared to HCNFs, although both of them had comparative oxidation potential. These results prove that our established Bi/HCNFs possess superior electrocatalytic activity towards the oxidation of NO_2_^−^ to NO_3_^−^, which can be used as a highly efficient electrocatalyst to construct an electrochemical sensor for NO_2_^−^ detection.

Subsequently, the parameters that may influence the response of Bi/HCNFs to NO_2_^−^, such as pH and applied potential, were thoroughly investigated. Figure S4 shows the electrochemical response of Bi/HCNFs toward nitrite (1 mmol·L^−1^) at different pH levels (from 3.0 to 10.0); we can see that the peak current of NO_2_^−^ oxidation presented a distinct decrease with pH increasing, and there was no obvious change in the current when the pH changed from 6.0 to 10.0 on the response of Bi/HCNFs to nitrite peak oxidation [35]. Hence, a PBS buffer solution with pH = 7.0 was selected as the supporting electrolyte in the following experiments, and such a pH value can be beneficial for applications in real food and environment samples. To find the optimal potential to apply to the electrochemical sensor in amperometric analysis, the amperometric current response of Bi/HCNFs with continuous added NO_2_^−^ was repeated at different potentials from 0.70 V to 0.85 V under stirring conditions (Figure S5). The results exhibited a gradually increased current response with the increase in applied potentials, but a bigger noise signal could be observed at a higher potential. Thus, 0.80 V was selected as the optimal potential in the following electrochemical tests.

To thoroughly understand the electrochemical behavior of NO_2_^−^ peak oxidation on Bi/HCNFs, the relationship between the oxidation peaks of NO_2_^−^ and the scan rates (from 10 to 100 mV s^−1^) was explored in 0.1 M PBS containing 1 mmol·L^−1^ NO_2_^−^. As shown in Figure 3b, the anodic peak current (I_p_, μA) increased with the gradually increasing scan rate (v, mV s^−1^), which was proportional to the square root of the scan rate; the corresponding equation could be simulated as I_p_ = 5.328ν^1/2^ + 1.411 (R^2^ = 0.9910) (Figure 3c). This result demonstrates that the electrochemical oxidation of nitrite on Bi/HCNFs is a diffusion-controlled irreversible process [14,36]. Additionally, the oxidation peak potential (E_p_) also showed a positive shift with the increasing scan rate, and the linear regression equation between E_p_ (V) and logv could be expressed as E_p_ = 0.04176logv + 0.7238 (R^2^ = 0.9893) (Figure 3d). Consequently, the number of electrons of this irreversible electrochemical process could be calculated through the following equation, which describes the linear relationship between E_p_ and logv (Equation (1)):

(1) $$E_{p}=\frac{2.303\mathrm{RT}}{2\left(1-\alpha \right)n_{\alpha }F}\mathrm{logv}+K\;$$

where n_α_ represents the number of electron transfer, α is the electro-transfer coefficient and R, T and F refer to the thermodynamic constant (8.314 J mol^−1^ K^−1^), thermodynamic temperature (298 K) and Faraday constant (96,485 C mol^−1^), respectively. α can be calculated to be 0.86 for Bi/HCNFs through the Tafel plot. According to the linear equation of E_p_ vs. logv, the value of n is calculated to be 0.933 ≈ 1, indicating that the one-electron transfer process of NO_2_^−^ oxidation on Bi/HCNFs is the rate-determining step [37]. Therefore, we speculated that there were three steps in the process of NO_2_^−^ oxidation on Bi/HCNFs: (i) NO_2_^−^ in the solution firstly bonded on the surface of Bi/HCNFs through a complex interaction to form [Bi/HCNFs(NO_2_^−^)] (Equation (2)); (ii) the complex [Bi/HCNFs(NO_2_^−^)] lost one electron to produce NO_2_ (Equation (3)); and (iii) NO_2_ molecules were involved in the disproportionation reaction and generated NO_2_^−^ and NO_3_^−^ (Equation (4)). Further electrocatalytic oxidation of NO_2_^−^ occurred to form NO_3_^−^, and thus NO_3_^−^ is the final possible product (Equation (5)). The detailed reaction process of NO_2_^−^ oxidation on Bi/HCNFs can be expressed as follows:

Bi/HCNFs + NO_2_^−^ → [Bi/HCNFs(NO_2_^−^)] (2)

Bi/HCNFs(NO_2_^−^) → Bi/HCNFs + NO_2_ + e (3)

2NO_2_ + H_2_O → NO_3_^−^ + NO_2_^−^ + 2H^+^ (4)

NO_2_^−^ + H_2_O → NO_3_^−^ + 2H^+^ + 2e^−^ (5)

### 2.3. Detection of Nitrite Based on Bi/HCNFs Electrode

**Figure 4:**
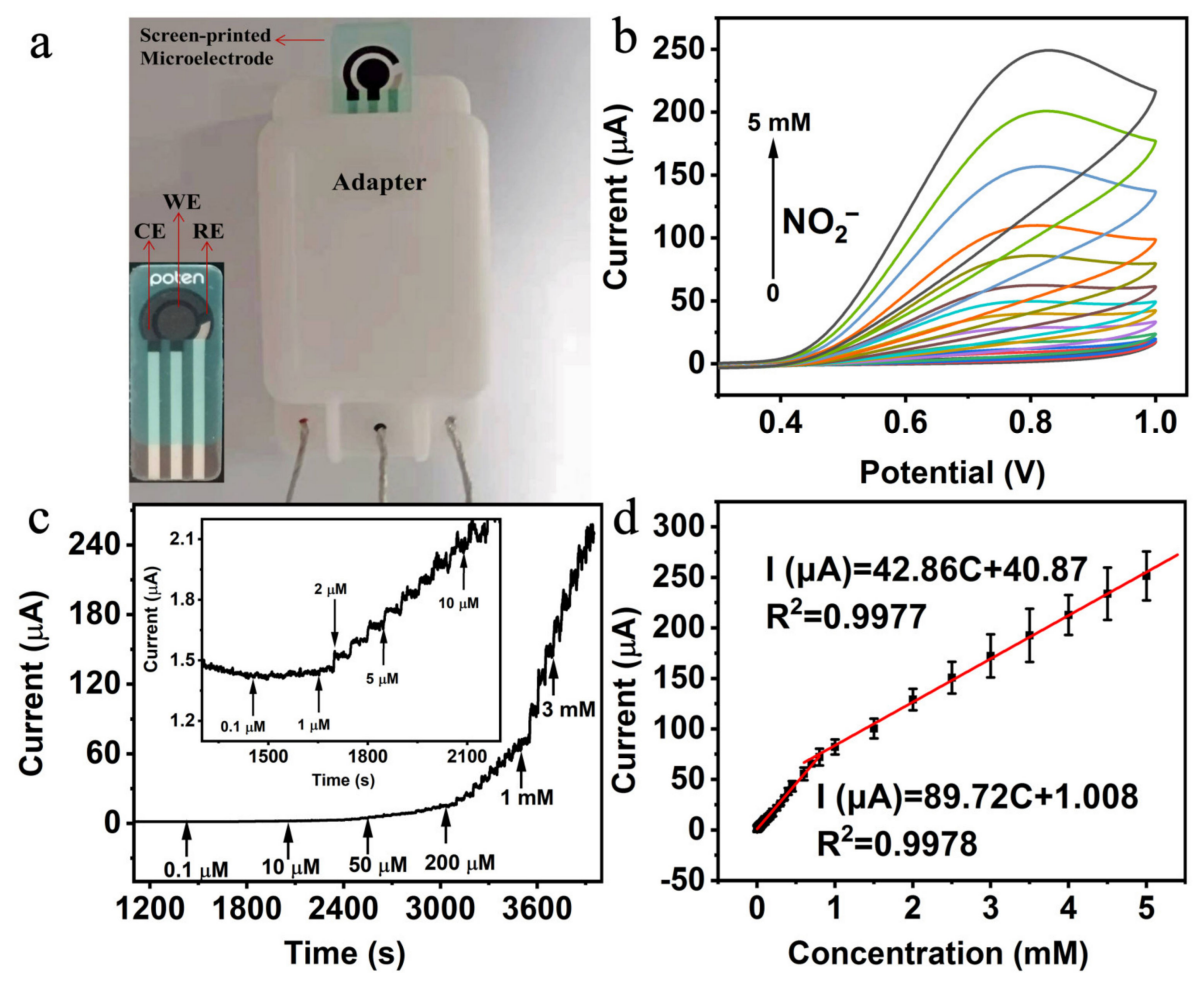
(**a**) The photo of the commercial screen-printed electrode. (**b**) CV curves of Bi/HCNFs-SPE in 0.1 mol·L^−1^ PBS (pH 7.0) with different concentrations of NO_2_^−^ ranging from 0 to 5 mmol·L^−1^. (**c**) Amperometric responses of Bi/HCNFs-SPE with the continuous addition of nitrite at 0.80 V vs. Ag/AgCl (inset: corresponding response at low concentration of NO_2_^−^). (**d**) Corresponding fitting curves of current response vs. nitrite concentration.

By locating Bi/HCNFs on the working electrode (WE) of the screen-printed electrode (SPE) with Ag/AgCl and carbon as the reference electrode (RE) and counter electrode (CE), respectively (Figure 4a), the modified electrode of Bi/HCNFs-SPE was constructed and then used for NO_2_^−^ detection. Such Bi/HCNFs-SPE possesses low cost, simple operation and high stability attributes, making it a potential sensing platform for NO_2_^−^ detection. The electrochemical performance of Bi/HCNFs-SPE was first investigated in 0.1 mol·L^−1^ PBS solution with the presence of 1 mmol·L^−1^ NO_2_^−^. As can be seen from Figure S6, an obvious oxidation peak was observed, and the peak current was about four-fold higher than that of bare SPE, indicating the higher sensitive response of Bi/HCNFs-SPE to NO_2_^−^. Figure 4b shows the CV curves based on Bi/HCNFs-SPE in 0.1 mol·L^−1^ PBS (pH = 7.0) with different nitrite contents (from 0 to 5 mmol·L^−1^). We could see that the current increased gradually with the increase of nitrite concentration from 0 to 5 mmol·L^−1^. To further evaluate the electrochemical response of Bi/HCNFs-SPE toward NO_2_^−^, chronoamperometric testing was recorded with the consecutive addition of various NO_2_^−^ under stirring constantly at 0.80 V vs. Ag/AgCl. As shown in Figure 4c, a current response could be observed even with the addition of lower concentrations of 0.1 μmol L^−1^ NO_2_^−^, which increased with the increasing concentration of NO_2_^−^. The current could become steady within 3 s of the addition of NO_2_^−^, indicating the fast amperometric response of Bi/HCNFs-SPE towards NO_2_^−^. Figure 4d displays the corresponding calibration curve from Figure 4c, and a gradually increased current (I, μA) with the increasing NO_2_^−^ concentration (C, mM) can be observed. In the higher concentration range (0.8~5 mmol·L^−1^), a corresponding linear equation can be expressed as I = 42.86C + 40.87 (R^2^ = 0.9978) with a sensitivity of 634.9 μA mM^−1^ cm^−2^ and a detection limit of 38 nmol·L^−1^. In the range of 0.1~800 μmol·L^−1^, a linear equation could be calculated as I = 89.72C + 1.008 (R^2^ = 0.9978) with an excellent sensitivity of 1269.9 μA mM^−1^ cm^−2^, and the corresponding detection limits could be calculated to be 19 nmol·L^−1^ with a signal-to-noise ratio of 3. These results declared that our established Bi/HCNFs-SPE possessed higher electrochemical properties regarding NO_2_^−^ than most previously reported metal-based electrochemical sensors; see Table S1 [38,39,40,41,42,43,44,45,46].

**Figure 5:**
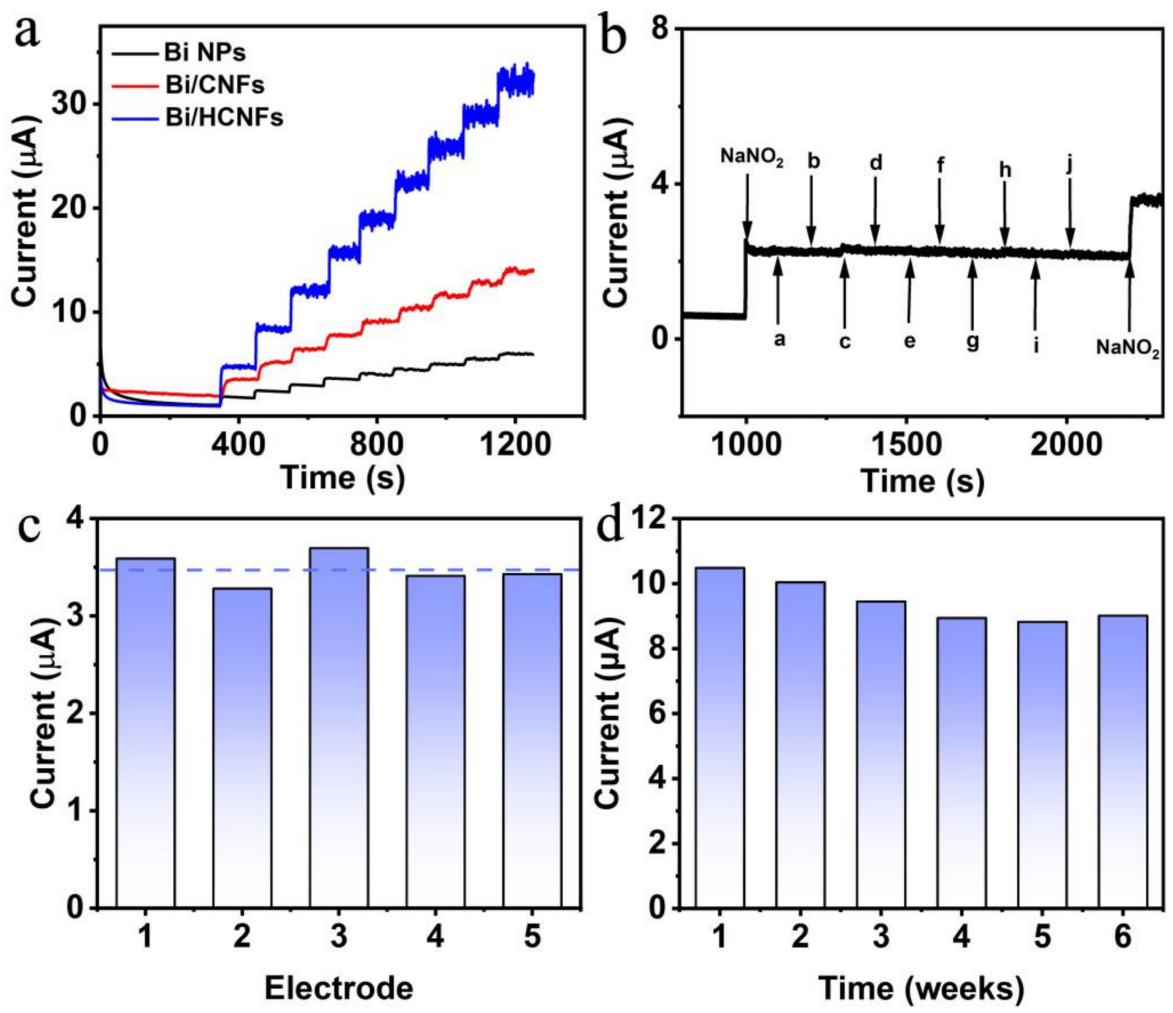
(**a**) The amperometric responses of different electrocatalysts modifying GCE with the successive addition of 0.2 mmol L^−1^ nitrite at 0.80 V vs. Ag/AgCl. (**b**) Amperometric responses of Bi/HCNFs-SPE to the successive addition of 0.1 mmol L^−1^ NO_2_^−^, 1 mmol L^−1^ Na_2_CO_3_ (a), Na_2_SO_4_ (b), K_2_CO_3_ (c), NaCl (d), Glu (e), sucrose (f), NaNO_3_ (g), citric acid (h), sodium benzoate (i), benzoic acid (j), and 0.1 mmol L^−1^ NO_2_^−^ in 0.1 mol L^−1^ PBS at 0.80 V vs. Ag/AgCl. (**c**) The amperometric responses towards 0.1 mmol L^−1^ nitrite using five newly prepared Bi/HCNFs-SPE in stirred 0.1 mmol L^−1^ PBS solution (pH 7.0); (**d**) The amperometric responses towards 0.2 mmol L^−1^ nitrite at Bi/HCNFs-SPE with an interval of one week.

We speculated that these excellent electrochemical properties of Bi/HCNFs-SPE to NO_2_^−^ should derive from the special honeycomb-like porous structure of HCNFs, which not only leads to the high surface area of HCNFs, but also to the uniformly distributed Bi NPs with smaller size. To demonstrate this, the electrochemical response of Bi NPs loaded on CNFs without a porous structure (Bi/CNFs) and Bi NPs without a substrate to NO_2_^−^ were also investigated as a comparison. Figure 5a displays the electrochemical response of Bi/HCNFs, Bi/CNFs and Bi NPs to NO_2_^−^, respectively. The higher response of Bi/HCNFs to nitrite than that of Bi NPs and Bi/CNFs demonstrated the higher catalytic activity of Bi/HCNFs. Based on this, the nitrogen adsorption–desorption isotherm of HCNFs was performed and a mass of mesopores and macropores could be seen from the pore size distribution, which led to the high porosity of HCNFs (Figure S7). In addition, the electrochemical active surface areas (ECSA) of Bi NPs, Bi/CNFs and Bi/HCNFs were compared by measuring the capacitance of the electric double layer (Figure S8a–c), and the result (Figure S8d) indicated that Bi/HCNFs had the largest capacitance, of 1.42 mF cm^−2^, which was 3.3 and 8.6 times than that of Bi/CNFs (0.43 mF cm^−2^) and Bi NPs (0.165 mF cm^−2^), respectively. Therefore, all the experiment results above-mentioned confirmed that the high specific area of HCNFs and the small size of Bi NPs endowed the excellent electrochemical performance of Bi/HCNFs for nitrite detection, further demonstrating its great potential applications for constructing electrochemical sensors with higher performance.

### 2.4. Interference, Reproducibility and Stability of Bi/HCNFs-SPE and Real Sample Analysis

To demonstrate its practical application, the interference, reproducibility and stability of the prepared Bi/HCNFs-SPE for NO_2_^−^ detection were then investigated in detail. As seen in Figure 5b, Bi/HCNFs-SPE exhibits a sharp increase in current response after the addition of NO_2_^−^ (0.1 mmol L^−1^). However, the addition of common anions/cations (Na^+^, K^+^, CO_3_^2−^, SO_4_^2−^, Cl^−^), as well as usually coexisting food additives (including glucose, sucrose, nitrate, citric acid, benzoic acid, sodium benzoate) did not influence the detection of NO_2_^−^, even with a concentration ten times higher than nitrite. The further addition of NO_2_^−^ (0.1 mmol L^−1^) in the above mixture could also result in a comparable current increasing with the first time added, indicating the excellent selectivity of Bi/HCNFs-SPE to nitrite.

Reproducibility and stability are other key parameters for practical applications of sensors. Figure 5c indicates the responses of five constructed Bi/HCNFs-SPE regarding the NO_2_^−^ response (0.1 mmol L^−1^), and the slight variations with a relative standard deviation (RSD) of 4.6% indicated the good reproducibility of the modified electrode based on Bi/HCNFs. Furthermore, the long-term stability of Bi/HCNFs-SPE for nitrite detection was also tested. As shown in Figure 5d, the response current could be maintained at 86% after five weeks of continuous testing, indicating its desirably long stability in practical applications.

The analytical applicability of fabricated Bi/HCNFs-SPE was investigated by monitoring the nitrite concentration in real food and environmental samples. As listed in Table 1, satisfactory recoveries of the electrochemical strategy based on the amperometric method in the range of 94–105% and with an RSD from 0.4% to 4.9% were obtained with different spiking concentrations of nitrite in tap water, pickles and sausage. To further prove the accuracy of the results obtained from Bi/HCNFs-SPE, the UV–vis spectrophotometric method was also adopted to determine the nitrite content in the same spiked samples, and the comparable results indicated the reliability of the constructed Bi/HCNFs-SPE for practical applications in complex samples. More interestingly, based on the small size of the as-prepared electrode, our well-fabricated disposable SPE could also be used for nitrite detection in one drop of aqueous solution through the cyclic voltammetry method (about 200 μL, Figure S9), with good recoveries between 102 and 106% (Table S2). These excellent properties of Bi/HCNFs-SPE, accompanied by the advantages of low-cost, convenient operation and quick response, demonstrate its potential application in complicated food and environment samples which contain nitrite.

## 3. Materials and Methods

### 3.1. Reagent and Materials

Bismuth nitrate pentahydrate (Bi(NO_3_)_3_·5H_2_O), hydrazine hydrate (N_2_H_4_·H_2_O) and ammonia (NH_3_·H_2_O) were bought from Chengdu Kelong Chemicals (Chengdu, China), polyvinyl alcohol (PVA, MW~67,000) and polytetrafluoroethylene (PTFE, 120 nm) were obtained from Aladdin Reagent Co., Ltd. (Shanghai, China), and glucose (Glu), sucrose, urea, sodium chloride (NaCl), sodium carbonate (Na_2_CO_3_), sodium sulfate (Na_2_SO_4_), potassium carbonate (K_2_CO_3_), citric acid, benzoic acid, sodium benzoate, sodium nitrite (NaNO_2_), sodium nitrate (NaNO_3_), sodium dihydrogen phosphate (NaH_2_PO_4_·2H_2_O) and disodium hydrogen phosphate (Na_2_HPO_4_·12H_2_O) were purchased from Aladdin Chemical Reagent Company (Shanghai, China). All the above chemicals were analytical grade and did not undergo further purification before use.

### 3.2. Characterizations

X-ray diffraction patterns were obtained with a Shimazu XRD-6100 diffractometer with Cu Kα radiation (40 kV, 30 mA) of wavelength 0.154 nm (Kyoto Japan). Scanning electron microscopy (SEM) images were collected on GeminiSEM 300 from Carl Zeiss Microscopy GmbH (Oberkochen, Germany). Energy-dispersive X-ray spectroscopy (EDS) was recorded on a Gemini SEM 300 scanning electron microscope (ZEISS; Oberkochen, Germany) at an accelerating voltage of 5 kV. Transmission electron microscopy (TEM) images were obtained using an H-800 electron microscope and an H-8010 scanning system (Hitachi, Japan). X-ray photoelectron spectroscopy (XPS) data were measured on Thermo Scientific K-Alpha with Al Kα radiation. The zeta potential was conducted on a Malvern Nano ZS90 Zetasizer Nano instrument (Worcestershire, UK). The ultraviolet–visible (UV–Vis) absorption spectra were received on a UV-2900 spectrophotometer.

### 3.3. Synthesis of HCNFs

PVA was dissolved in the deionized water to prepare the PVA solution (15 wt.%) at 60 °C for 3 h with a constant stirring rate. After cooling to room temperature, the PTFE water emulsion (60 wt.%) was added to the above mixture solution and kept stirring vigorously for 3 h. Subsequently, the precursor solution was put in a plastic syringe with a stainless steel nozzle connected to a high-voltage power supply. The electrospinning process was performed with a high voltage of 18 kV and an 18 cm distance between the needle and the aluminum foil collector. After that, the as-spun film was kept in a muffle furnace at 280 °C for 3 h and then calcined at 800 °C at Ar atmosphere with a heating rate of 5 °C min^−1^ to collect as-fabricated HCNFs nanofibers. For comparison, carbon nanofibers without pores (CNFs) were synthesized with the same procedures, except for the use of PTFE.

### 3.4. Synthesis of Bi/HCNFs

The Bi/HCNFs were prepared using a facile hydrothermal method. Typically, HCNFs (1 mg mL^−1^) were first dispersed in deionized water and then added to Bi(NO_3_)_3_ (0.5 mmol·L^−1^) with continuous stirring for 3 h. Next, 3 mL N_2_H_4_·H_2_O (85 wt%) and 1 mL NH_3_·H_2_O (28 wt%) were injected into the above mixture and kept magnetically stirring. After that, the suspension was carefully transferred into a 50 mL Teflon-lined autoclave, which was sealed and heated at 120 °C for 18 h. Subsequently, the as-produced product was thoroughly washed several times with deionized water and acetone, respectively. Finally, the collected black powder was dried at 40 °C under a vacuum. However, Bi/CNFs were prepared by using CNFs instead of HCNFs under the same procedure. For comparison, Bi NPs and HCNFs were synthesized with a similar procedure without HCNFs and Bi(NO_3_)_3_ used.

### 3.5. The Pretreated Actual Samples and Spectrophotometric Test of Nitrite

The tap water was acquired from Sichuan University and was filtered with a 0.22 μm membrane before testing. The pickle and sausage were purchased from a local market. Pickles were firstly crushed into homogenate and 5 g of the above sample was dissolved into 50 mL ultrapure water and centrifuged to acquire the supernatant. The sausage was ground into puree and a part of it (5 g) was added into a 150 mL conical flask with the addition of 80 mL ultrapure water, which was subjected to ultrasound for 30 min. Subsequently, the mixture was heated at 75 °C and then cooled to room temperature. Finally, the turbid solution was centrifuged to obtain the supernatant at 10,000 r/min. According to the Chinese National Food Safety Standard (GB 5009.33-2016), the food samples were pretreated with 0.1 mol L^−1^ PBS solution (pH 7.0) before measuring.

As a comparison, the nitrite concentrations of real samples were quantified through the spectrophotometric method. Generally, sulfonamide diazide coupled with N-(1-naphthalene)-ethylenediamine dihydrochloride forms a purple azo dye at a pH of 2.0~2.5. The color agent was prepared by mixing 0.2 g N-(1-naphthyl) ethylenediamine dihydrochloride, 2.0 g sulfonamide, and 5.88 mL H_3_PO_4_ in 100 mL ultrapure water. After that, 1 mL of spiked sample was reacted with the mixture of 1 mL color agent and 2 mL ultrapure water under dark conditions. This could be quantified by photometric measurements in the wavelength of 400~700 nm. According to the absorption spectrum, 546 nm was selected as the maximum absorption wavelength to determine nitrite content.

### 3.6. Electrochemical Properties Investigation of Bi/HCNFs

The electrochemical properties of Bi/HCNFs were investigated by immobilizing Bi/HCNFs on a glassy carbon electrode (GCE) with a diameter of 3 mm from Shanghai Yuehe Electronic Technology Co., LTD. The screen-printed electrode was purchased from Qingdao Bonaco Technology Co., Ltd. (Qingdao, China) (POTE, TC201). The blank GCE was firstly polished with α-Al_2_O_3_ powder (0.05 μm) and then subjected to ultrasound with ethanol and deionized water, respectively, before the modification or direct use. The prepared Bi/HCNFs were dispersed in an ethanol aqueous solution (ethanol/water = 3:1) under ultrasonication to form an aqueous suspension (5 mg mL^−1^), and then 5 μL of the resulting suspension was dropped on the pretreated GCE surface. For comparison, Bi/CNFs, Bi NPs and HCNFs-modified GCE were prepared with the above process under the same modification quantities. The cyclic voltammetry and chronoamperometry curves were measured using a CHI 660E electrochemical analyzer (CHI Instruments, Inc., Shanghai, China) with a three-electrode system, with Pt wire as the counter electrode and Ag/AgCl/saturated KCl solution as the reference electrode. The electrolyte solution was 0.1 M PBS solution (pH 7.0).

## 4. Conclusions

In summary, honeycomb-like carbon nanofibers (HCNFs) were successfully fabricated with an electrospun method, and the obtained HCNFs can be used as desired substrates for Bi nanoparticle decoration (Bi/HCNFs). The as-fabricated Bi/HCNFs exhibited excellent electrochemical properties towards NO_2_^−^ oxidation, and therefore they can be used for the construction of electrochemical sensors for NO_2_^−^ detection by locating Bi/HCNFs on a commercial screen-printed electrode (Bi/HCNFs–SPE), which has been successfully used for NO_2_^−^ analysis in complex samples with high sensitivity, excellent selectivity, good anti-interference and satisfactory stability. The high properties of the sensor should be attributed to the unique structure of HCNFs, which can not only enhance the active area of material, but also regulate the growth of Bi nanoparticles, leading to highly distributed smaller Bi NPs on HCNFs. Notably, the constructed sensor based on Bi/HCNFs can also be used for NO_2_^−^ detection in sausage, pickles and tap water samples, even in one drop of sample. The obtained satisfactory recoveries for NO_2_^−^ detection indicate that Bi/HCNFs–SPE can be used as a promising sensing platform for the accurate analysis of nitrite in the fields of environment and food.

## Figures and Tables

**Table 1:** Analysis results of real samples conducted utilizing the modified electrode and spectrophotometric method.

Electrochemical Method					Spectrophotometric Method		
Samples	Added (μM)	Found (μM)	Recovery (%)	^a^ RSD (%)	Found (μM)	Recovery (%)	^a^ RSD (%)
Tap water	5.0 50.0 500.0	4.9 52.1 527.1	98.0 104.2 105.4	0.4 0.8 1.9	4.8 46.7 501.6	96.0 93.4 100.3	2.8 1.6 0.5
Pickles	5.0 50.0 500.0	4.9 50.5 510.5	98.0 101.0 102.1	0.5 0.8 2.1	5.3 49.6 508.6	106.0 99.2 101.7	2.4 1.1 0.1
Sausage	5.0 50.0 500.0	4.9 47.1 511.5	98.0 94.2 102.3	4.9 1.4 4.8	5.1 50.8 513.3	102.0 101.6 102.7	1.4 1.5 0.7

Bulleted li ^a^ RSD: Relative standard deviation.

## Data Availability

The data presented in this study are available in the Supplementary Materials.

## Supplementary Materials

The following supporting information can be downloaded at: https://www.mdpi.com/article/10.3390/molecules28093881/s1, Figure S1: particle size distribution histograms of Bi NPs on the surface of HCNFs; Figure S2: STEM image and corresponding elemental mapping images of Bi, C, O for Bi/HCNFs, respectively; Figure S3:The SEM image of Bi NPs (inset of Figure S3: high magnification SEM image); Figure S4: The diagram of CV peak current and pH in the presence of 1 mM NO_2_^−^ ranging from 6.0 to 10.0; Figure S5: The amperometric responses of Bi/HCNFs at different potentials of 0.70–0.85 V vs. Ag/AgCl with successive additions of 0.2 mM nitrite; Figure S6: CV responses of disposable bare SPE and Bi/HCNFs modified SPE in 0.1 M PBS (pH 7.0) with the presence of 1 mM NO_2_^−^; Figure S7: (a) Nitrogen adsorption–desorption isotherms of HCNFs and (b) its corresponding pore size distribution; Figure S8: CV curves of Bi NPs (a), Bi/CNFs (b) and Bi/HCNFs (c) at different scan rates from 20 to 120 mV s^−1^ in the region without faradic current. (d) Plot of corresponding capacitive current densities of Bi NPs, Bi/CNFs and Bi/HCNFs with scan rates; Figure S9: The photo of commercial screen-printed electrodes with a drop of solution containing nitrite (200 μL). Table S1: Comparison of electrocatalytic performance towards nitrite with previous electrochemical sensors; Table S2: Analysis results of trace nitrite in real samples on Bi/HCNFs-SPE through dropping tests (200 μL).

## Sample Availability Statement

Samples of the compounds are available from the authors.
