# Supplementary material for: Bismuth-Decorated Honeycomb-like Carbon Nanofibers: An Active Electrocatalyst for the Construction of a Sensitive Nitrite Sensor
Source: Molecules. 2023 May 4;28(9):3881. doi: 10.3390/molecules28093881 (PMC10180303; doi:10.3390/molecules28093881)
Supplement: Supplementary file 1 [file molecules-28-03881-s001.zip › molecules-2382072-supplementary.pdf]

Supplementary Material

Bismuth Decorated Honeycomb-like Carbon Nanofibers: An Active Electrocatalyst for Construction of Sensitive Nitrite Sensor

Fengyi Wang [1,2], Ye Li [2], Chenglu Yan [3], Qiuting Ma [2], Xiaofeng Yang [1,*], Huaqiao Peng [3], Huiyong Wang [4], Juan Du [2,*], Baozhan Zheng [2,*] and Yong Guo[2]

[1] Institute of Quality Standard and Testing Technology for Agro-products of Sichuan Academy of Agricultural Sciences, Chengdu 610066, China.
[2] College of Chemistry, Sichuan University, No. 29 Wangjiang Road, Chengdu 610064, China.
[3] The Second Research Institute of Civil Aviation Administration of China, Chengdu 610041, China.
[4] College of Chemistry and Chemical Engineering, Henan Normal University, Henan 453002, China.
* Correspondence: yangxiaofeng_cd@sina.com (X. Yang); dujuanchem@scu.edu.cn (J. Du); zhengbaozhan@scu.edu.cn (B. Zheng).

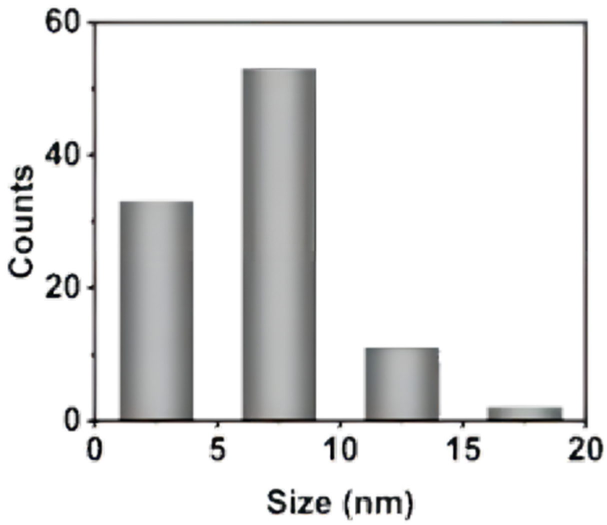

Figure S1. particle size distribution histograms of Bi NPs on the surface of HCNFs.

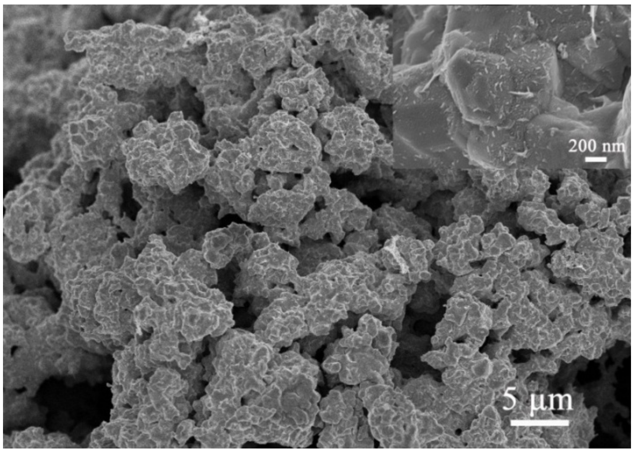

Figure S2. The SEM image of Bi NPs (inset of Figure S2 high magnification SEM image).

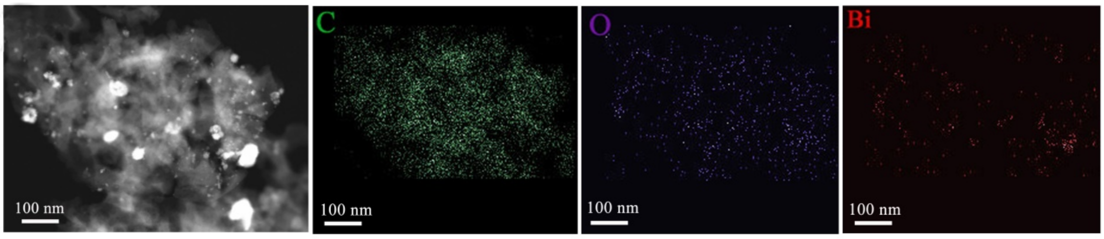

Figure S3. STEM image and corresponding elemental mapping images of Bi, C, O for Bi/HCNFs, respectively.

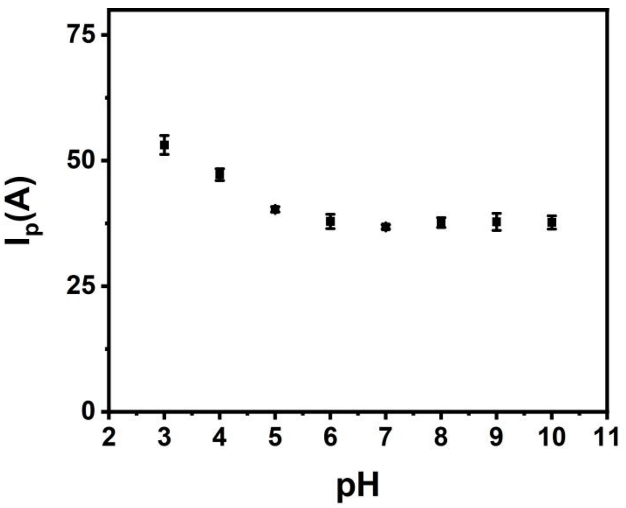

Figure S4. The diagram of CV peak current and pH in the presence of 1 mM NO_2^- ranging from 3.0 to 10.0.

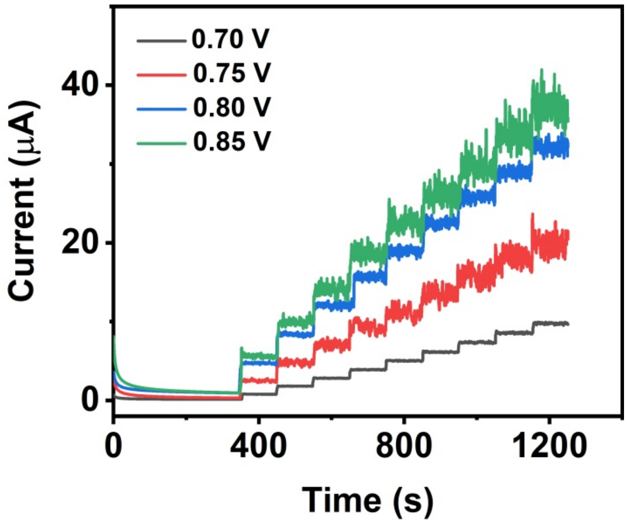

Figure S5. The amperometric responses of Bi/HCNFs at different potentials of 0.70-0.85 V vs. Ag/AgCl with successive additions of 0.2 mM nitrite.

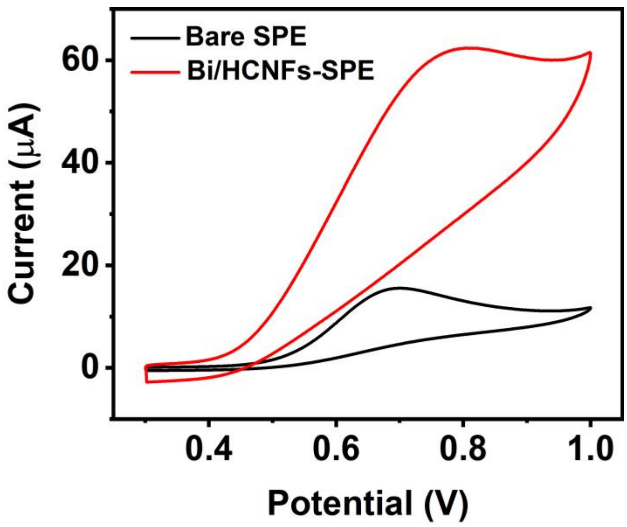

Figure S6. CV responses of disposable bare SPE and Bi/HCNFs modified SPE in 0.1 M PBS (pH 7.0) with the presence of 1 mM NO_2^-.

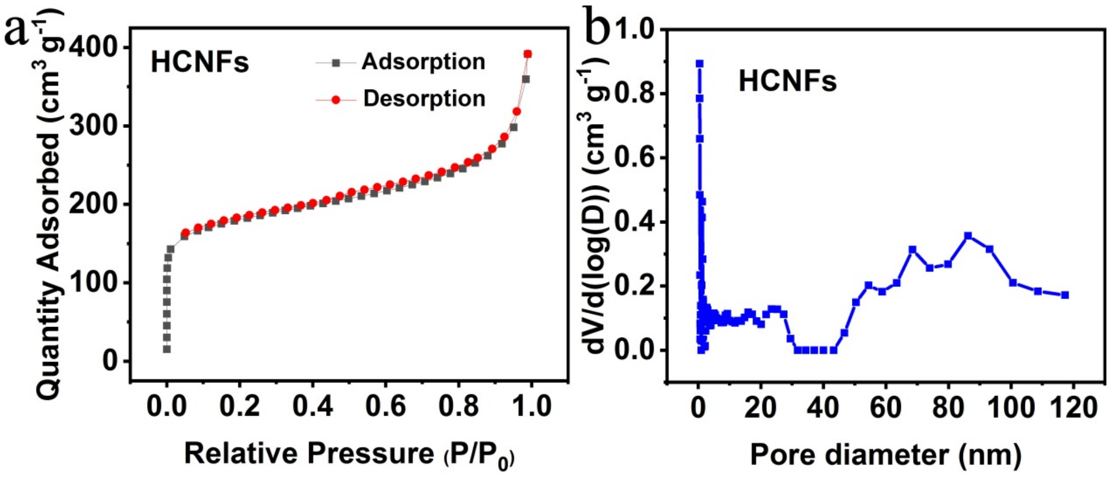

Figure S7. (**a**) Nitrogen adsorption-desorption isotherms of HCNFs and (**b**) its corresponding pore size distribution.

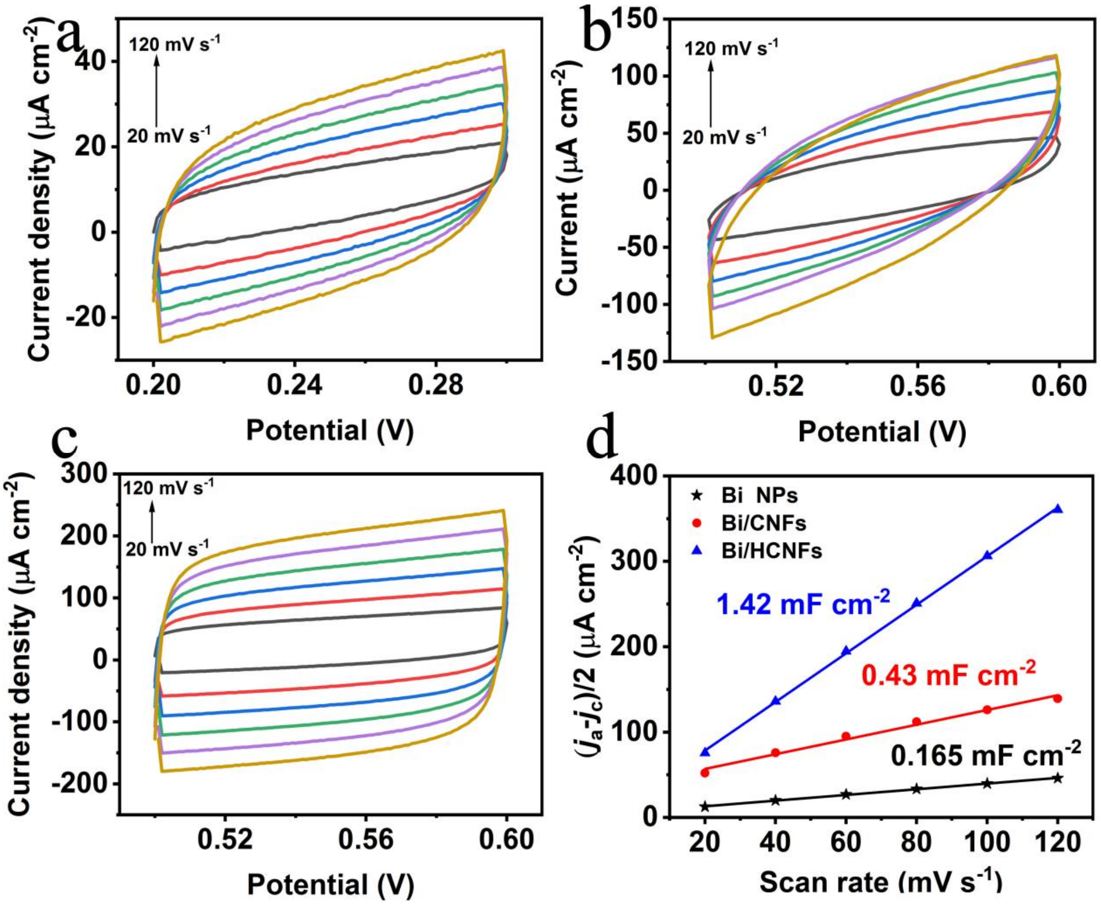

Figure S8. CV curves of Bi NPs (**a**), Bi/CNFs (**b**) and Bi/HCNFs (**c**) at different scan rates from 20 to 120 mV s^{-1} in the region without faradaic current. (**d**) Plot of corresponding capacitive current densities of Bi NPs, Bi/CNFs and Bi/HCNFs with scan rates, j_a and j_c are the current densities of the anodic and cathodic branch of CV at a defined potential, respectively.

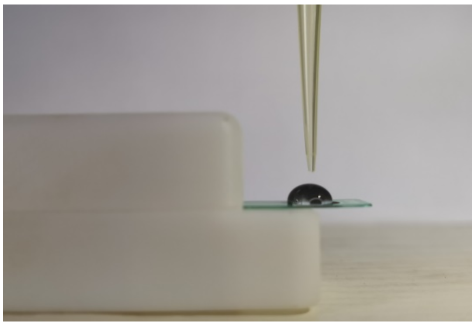

Figure S9. The photo of commercial screen-printed microelectrode with a drop of solution containing nitrite (200 μL).

Table S1. Comparison of electrocatalytic performance toward nitrite with previous electrochemical sensors.

Electrode materials	LOD (μM)	Sensitivity (μA mM^{-1} cm^{-2})	Reference
$NiFe_2O_4$ NPs	0.1236	7961.7	[1]
NiFe-LDH NSAs/CC	0.02	803.6	[2]
Cu-MOF/Au	0.082	252	[3]
Ag/HNTs/MoS_2	0.7	89.9 μA/mM	[4]
SnO_2/Pt/Ti/SiO_2	1.7	22.56 μA/mM	[5]
α-Fe_2O_3/CNTs hybrids	0.15	334	[6]
Fe_3O_4/rGO	0.3	226	[7]
Cu/MWNTs	1.8	455.84	[8]
TMPyPcCo/aCNTs	0.071	8 μA/mM	[9]
Bi/HCNFs	0.019	1269.9	This work

Table S2. Analysis results of trace nitrite in real samples on Bi/HCNFs-SPE through dropping tests (200 μL).

Samples	Added (μM)	Found (μM)	Recovery (%)	[a]RSD(%)
Tap water	500	526.8	105.4	0.89
	1000	1038	103.8	1.62
Pickles	500	514.7	102.9	0.34
	1000	1059	105.9	0.78
Sausage	500	515.3	103.0	1.71
	1000	1053	105.3	0.76

[a]RSD: Relative standard deviation.

References

1. Nithyayini, K.N.; Harish, M.N.K.; Nagashree, K.L. Electrochemical detection of nitrite at $NiFe_2O_4$ nanoparticles synthesised by solvent deficient method. *Electrochim. Acta* **2019**, *317*, 701–710.
2. Ma, Y.; Wang, Y.; Xie, D.; Gu, Y.; Zhang, H.; Wang, G.; Zhang, Y.; Zhao, H.; Wong, P.K. NiFe-layered double hydroxide nanosheet arrays supported on carbon cloth for highly sensitive detection of nitrite. *ACS Appl. Mater. Interfaces* **2018**, *10*, 6541–6551.
3. Chen, H.; Yang, T.; Liu, F.; Li, W. Electrodeposition of gold nanoparticles on Cu-based metal-organic framework for the electrochemical detection of nitrite. *Sens. Actuators B: Chem.* **2019**, *286*, 401–407.
4. Ghanei-Motlagh, M.; Taher, M.A. A novel electrochemical sensor based on silver/halloysite nanotube/molybdenum disulfide nanocomposite for efficient nitrite sensing. *Biosens. Bioelectron.* **2018**, *109*, 279–285.
5. Lete, C.; Chelu, M.; Marin, M.; Mihaiu, S.; Preda, S.; Anastasescu, M.; Calderón-Moreno, J.M.; Dinulescu, S.; Moldovan, C.; Gartner, M. Nitrite electrochemical sensing platform based on tin oxide films. *Sens. Actuators B: Chem.* **2020**, *316*, 128102.
6. Wang, K.; Wu, C.; Wang, F.; Liu, C.; Yu, C.; Jiang, G. In-situ insertion of carbon nanotubes into metal-organic frameworks-derived α-Fe_2O_3 polyhedrons for highly sensitive electrochemical detection of nitrite. *Electrochim. Acta* **2018**, *285*, 128–138.
7. Teymourian, H.; Salimi, A.; Khezrian, S. Fe_3O_4 magnetic nanoparticles/reduced graphene oxide nanosheets as a novel electrochemical and bioeletrochemical sensing platform. B*iosens. Bioelectron.* **2013**, 49, 1–8.
8. Manoj, D.; Saravanan, R.; Santhanalakshmi, J. Agarwal, S.V. Gupta, K.; Boukherroub, R. Towards green synthesis of monodisperse Cu nanoparticles: An efficient and high sensitive electrochemical nitrite sensor. *Sens. Actuators B: Chem.* **2018**, *266*, 873–882.
9. Zhang, J.; Zhang, Z.; Wu, H.; Wu, F.; He, C.; Wang, B.; Wu, Y.; Ren, Z. An electrochemical bifunctional sensor for the detection of nitrite and hydrogen peroxide based on layer-by-layer multilayer films of cationic phthalocyanine cobalt(II) and carbon nanotubes. *J. Mater. Chem. B* **2016**, *4*, 1310–1317.
